# Psychometric Properties of the Trail Walking Test for People With Stroke

**DOI:** 10.3389/fneur.2022.821670

**Published:** 2022-03-03

**Authors:** Shamay S. M. Ng, Tai-Wa Liu, Joshua Tsoh, Peiming Chen, Tony S. Cheng, Marco C. H. Cheung, Anthony H. H. Leung, Liam L. Y. Ng, Ken Y. K. So, Mimi M. Y. Tse

**Affiliations:** ^1^Department of Rehabilitation Sciences, The Hong Kong Polytechnic University, Kowloon, Hong Kong SAR, China; ^2^School of Nursing & Health Studies, Hong Kong Metropolitan University, Kowloon, Hong Kong SAR, China; ^3^Department of Psychiatry, Prince of Wales Hospital and Shatin Hospital, Hong Kong, Hong Kong SAR, China; ^4^School of Nursing, The Hong Kong Polytechnic University, Kowloon, Hong Kong SAR, China

**Keywords:** trail walking test, dual tasking, stroke, outcomes, lower limb

## Abstract

**Objective:**

To investigate (i) the inter-rater and test–retest reliability of the trail walking test (TWT) and the minimum detectable change in the TWT completion time; (ii) the correlations between the TWT completion time and stroke-specific impairments; and (iii) the cutoff TWT completion time to distinguish between people with stroke and healthy older adults according to dual-tasking ambulation ability.

**Design:**

Cross-sectional study.

**Setting:**

University-based rehabilitation center.

**Participants:**

In total, 104 people with stroke and 53 healthy older adults.

**Main Outcome Measures:**

The TWT, the Fugl–Meyer Assessment of Lower Extremity (FMA-LE), the ankle muscle strength test, the limit of stability (LOS) test, the Berg Balance Scale (BBS), the Timed Up and Go test (TUG), and the Community Integration Measure (CIM).

**Results:**

The mean TWT completion time in subjects with stroke was 124.906 s. The TWT demonstrated excellent inter-rater reliability [intraclass correlation (ICC) = 0.999] and good test–retest reliability (ICC = 0.876) in people with stroke. The TWT performance demonstrated significant negative correlations with the FMA-LE scores (*r* = −0.409), LOS movement velocity (affected and unaffected sides; *r* = −0.320 and −0.388, respectively), and LOS endpoint excursion (affected and unaffected sides; *r* = −0.357 and −0.394, respectively); a significant positive correlation with the LOS reaction time (affected side; *r* = 0.256); a moderate negative correlation with the BBS scores (*r* = −0.72); and an excellent positive correlation with the TUG completion time (*r* = 0.944). The receiver operating characteristic curve analysis revealed that an optimal cutoff of 69.61 s for the TWT completion time had an outstanding diagnostic power to distinguish between people with stroke and healthy older adults (area under the curve = 0.919) with high sensitivity (88.5%) and specificity (83.0%).

**Conclusion:**

Results of our preliminary study demonstrated that the TWT is a reliable, valid, sensitive, and specific clinical test for evaluating dual-tasking ambulation ability in people with stroke aged 45 years or above and without cognitive impairments. It can differentiate the dual-tasking ambulation ability between people with stroke and healthy older adults.

## Introduction

Globally, 13.7 million new cases of stroke and more than 6.16 million-related deaths occur each year and stroke is the second leading cause of death worldwide (1). According to the United States Centers for Disease Control and Prevention, there were 7.8 million people with stroke in 2018. The lifetime cost of stroke per person, including inpatient care and rehabilitation programs, is estimated to be US$140,000 (2). The cerebrovascular disease not only places a heavy financial burden on the families of stroke survivors, but also causes motor disabilities and cognitive impairments in survivors, which worsens their walking ability and cognitive integration in activities of daily living.

The ability to perform motor–motor or motor–cognitive functions is vital for the reintegration of people with stroke into the community. These people may have an elevated risk of falling in crowded environments due to the problems they face with dual tasking, namely, executive function deficit and poor walking adaptability (3, 4). There are several walking tests that attempt to assess motor cognitive function, but all of them have limitations. For example, Liu et al. asked subjects to continuously subtract 3 from a randomized 3-digit number while walking (5). Although this assessment involves executive function, it does not reflect the cognitive tasks people encounter while commuting in daily life, which are usually more advanced and complex. Because a major aim of stroke rehabilitation is to enable stroke survivors to reintegrate into the community, it is crucial to assess the motor–cognitive functions of these survivors. To that end, clinicians need a reliable and valid measure that establishes motor–cognitive functions of patients at baseline to monitor their progress as a result of treatment.

Gait control is a motor–cognitive task that demands a high level of walking adaptability and executive function to plan, adapt, update, and control simultaneously (6). It can be assessed using the trail walking test (TWT), which has been modified from the trail making test (TMT), a standard neuropsychological measure wherein subjects sequentially connect numbered circles on paper (7). The TWT has been proposed to assess dual motor–cognitive functions by instructing subjects to walk past 15 numbered plastic cones in ascending order (3). However, the psychometric properties of the TWT have not been investigated in people with stroke.

As a major aim of stroke rehabilitation is to optimize the performances of motor tasks of patients in everyday life, which requires the ability to perform motor–cognitive functions, it is valuable to explore a test that captures performance in both the motor and cognitive domains. Clinicians need a reliable and valid measurement that will establish the ability of patients to perform motor–cognitive functions at baseline, to monitor the progress of patients as a result of stroke rehabilitation. Thus, this study aimed to: (i) establish the inter-rater and test–retest reliability of the TWT; (ii) investigate the correlation of TWT performance with stroke-specific impairments; (iii) identify the minimal detectable change (MDC) in the TWT completion time; and (iv) determine the cutoff TWT completion time that may help differentiate between people with stroke and healthy older adults according to their dual-tasking ability.

## Materials and Methods

### Study Design

This was a cross-sectional study. The study objectives and assessment procedures were explained to all the subjects. A written informed consent was obtained from all the subjects before starting this study. Ethical approval was obtained from the Ethics Committee of the local institution. This study was conducted according to the guidelines of the Declaration of Helsinki.

### Sample Size Calculation

No previous study has investigated the reliability of TWT in people with stroke. Good test–retest reliability of TWT [intra-class correlation (ICC) = 0.945] was found in frail community-dwelling older adults (3). Assuming that an ICC value for assessing test–retest reliability in stroke survivors was 0.9, a sample size of ≥46 subjects was required to achieve 80% power to detect an ICC of 0.9 with the null hypothesis of ICC equal to 0.8 and a significance level of 0.05. The sample size estimation was conducted with the Power Analysis & Sample Size (PASS) software (Version Q21 14NCSS, LLC, Kaysville, Utah, USA). In order to make a more robust conclusion, total 69 subjects with stroke were recruited for assessing the reliability of TWT.

No previous study has investigated the correlation between TWT and stroke-specific outcome measures in people with stroke. Assuming a significant weak correlation (ρ = 0.25) exists between TWT and the stroke-specific outcome measures, a sample size of ≥95 subjects was required to achieve 80% of power and a significant level of 0.05. The sample size estimation was conducted with the software G^*^Power 3.1.9.7 (Franz Faul, University of Kiel, Kiel, Germany). In order to make a more robust conclusion, we increased the sample size to 104 to assess the correlation between TWT and the stroke-specific outcome measures.

### Participants

**Figure 1:**
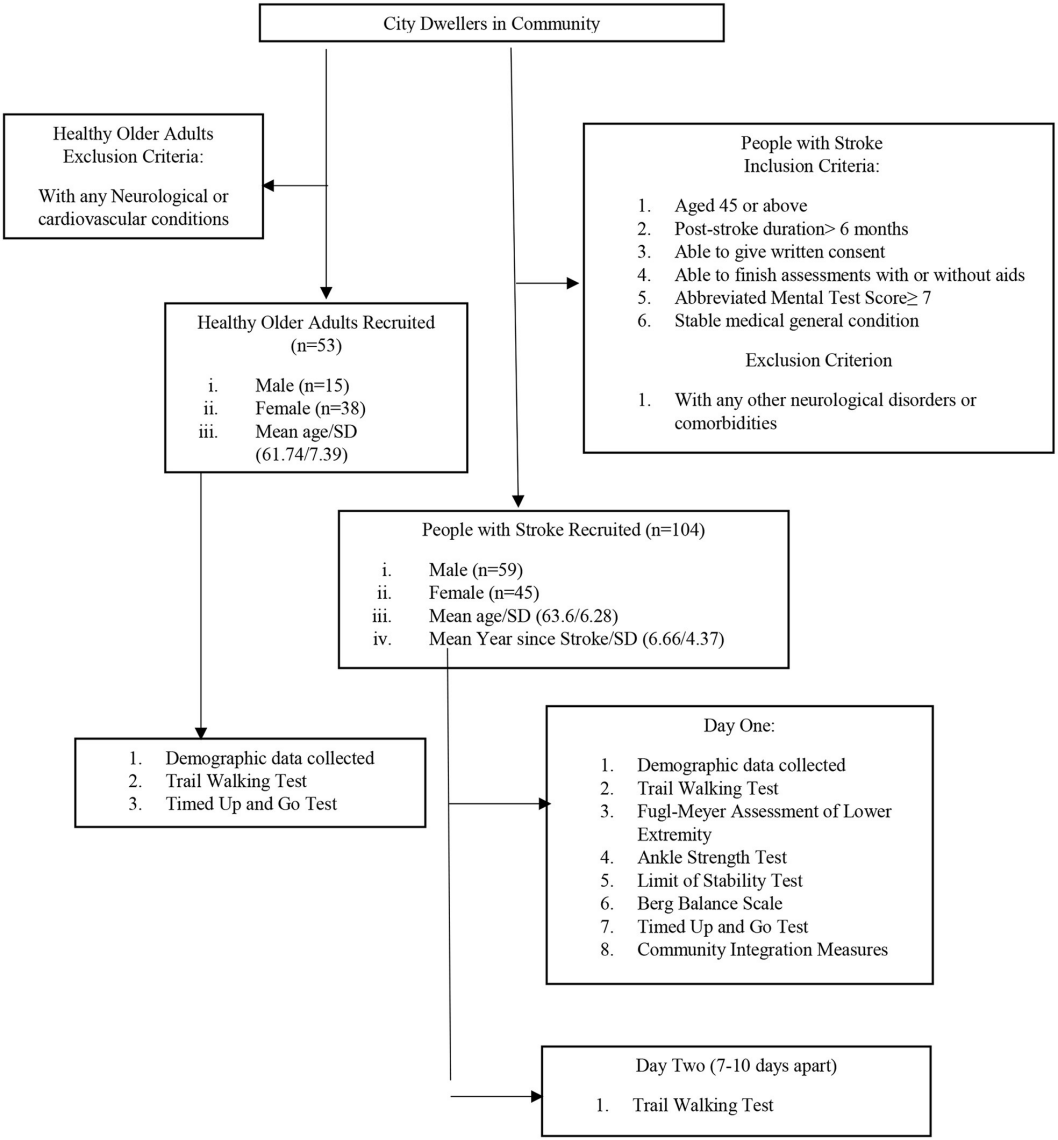
Flowchart of the study.

A total of 104 people with stroke (59 male, 45 female) were recruited from a local rehabilitation network through poster advertisements (see Figure 1). Subjects were included if: (i) they were aged 45 years or above; (ii) their poststroke duration was >6 months; (iii) they were able to give a written consent; (iv) they were able to finish the assessments independently with or without aids; (v) they had an Abbreviated Mental Test Score of ≥7; and (vi) they had a stable general medical condition. Subjects were excluded if they had any neurological disorder or comorbidity other than stroke such as Parkinson's disease, uncontrolled diabetes, or uncontrolled cardiovascular or musculoskeletal conditions that might hinder proper assessment.

A total of 53 healthy older adults (15 male, 38 female) older than 45 years with a stable health condition were recruited as the control group (see Figure 1). People with any neurological or cardiovascular disease that might affect proper assessment were excluded.

### Testing Procedures

To investigate the test–retest reliability, two trials of the TWT were conducted 7–10 days apart to minimize the learning effect of recurrent testing and physical changes in the subjects. Two raters (A and B) were trained to conduct the TWT in a standardized manner before the assessment and the TWT completion time was measured simultaneously by the two raters with digital stopwatches.

On day 1, the demographic data of all the 104 subjects with stroke were collected and the TWT was implemented. The subjects completed the Fugl–Meyer Assessment of Lower Extremity (FMA-LE), the ankle strength test, the limit of stability (LOS) test, the Berg Balance Scale (BBS), the Timed Up and Go test (TUG), and the Community Integration Measure (CIM) in a randomized order. The ankle strength test and TUG were conducted twice and their mean values were used in data analysis. Only one trial each was conducted of the TWT, FMA-LE, LOS test, BBS, and CIM. On day 2, 69 subjects with stroke among those 104 subjects who participated in day 1 assessment were randomly selected to perform the TWT on day 2 for assessing test–retest reliability.

Healthy older adults only performed the TWT and TUG on day 1 and their data were used to determine the cutoff scores for people with stroke. The known-group validity of the TWT was demonstrated by the ability of the TWT completion time to distinguish between people with stroke and healthy older adults.

### Outcome Measures

**Figure 2:**
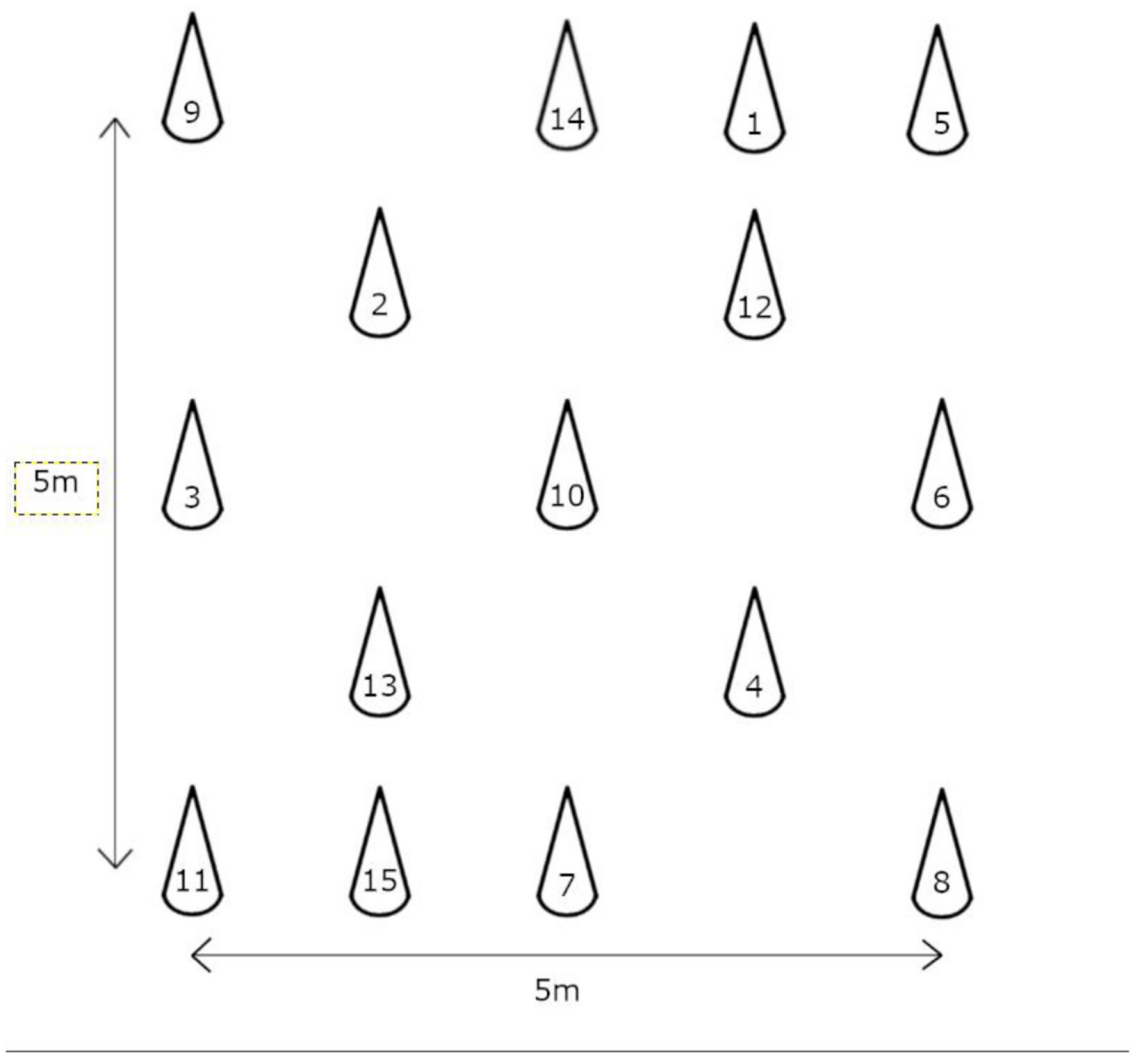
Trail Walking Test (TWT) setup.

In the TWT, both the cognitive and motor functions of the subjects are assessed simultaneously by having them walk past 15 numbered plastic cones sequentially at their normal walking speed (3). The setup and standardized instructions described by Yamada and Ichihashi were used in this study (3). Briefly, a grid with an area of 5 m^2^ was setup and the numbered cones were positioned randomly in the grid as shown in Figure 2. Two trained raters measured the completion time of subjects with stopwatches. Subjects who approached the wrong cone were reminded to return to the previous cone before searching for the next consecutive numbered cone. Only one test trial was conducted to prevent invalidation of results due to the learning effect of repeated trials within a session. The TWT was repeated 7 days after the first trial to determine the test–retest reliability. The TWT was shown to have excellent test–retest reliability (ICC = 0.95–0.99) in community-dwelling older adults, as well as a good correlation with the TMT (*r* = 0.458–562, *p* < 0.001) and a fair correlation with the TUG (*r* = 0.458–562, *p* = 0.001) (3).

The FMA-LE is a common assessment tool for evaluating poststroke motor function impairment in the lower extremities (8). It comprises 17 items assessing reflex, synergistic and isolated movements, and coordination and each item is scored on a 3-point scale from 0 to 2, with a maximum possible score of 34 (9). In people with stroke, the FMA-LE has been found to have excellent intrarater reliability (ICC = 0.93) and good test–retest reliability (ICC = 0.868). This tool has also yielded moderate to good concurrent validity with the motor assessment scale (*r* = 0.725) and the BBS (*r* = 0.661) in people with stroke (10).

The Nicholas handheld dynamometer (model 01160; Lafayette Instrument Company, Lafayette, Indiana, USA) was used to measure the maximum isometric voluntary contraction (MIVC) of both the affected and unaffected ankle dorsiflexors and plantar flexors. The hand-held dynamometer was positioned anteriorly or posteriorly over the heads of the first to fifth metatarsal bones to measure the strength of the ankle dorsiflexors and plantar flexors, respectively. Subjects were placed in the supine position and asked to perform the MIVC for 3 s. Each muscle group was tested twice by the same rater, with at least 30 s of rest between the two trials to reduce the effects of fatigue. The averages of the MIVC in kilograms were used in statistical analysis. The dynamometer used in the trials was shown to have excellent inter-rater reliability (ICC = 0.91) (11) and test–retest reliability (ICC = 0.93–1) (12) in community-dwelling older adults.

The LOS test is an assessment tool for measuring the outermost ranges of a ability of the person to move their center of pressure (COP) without changing the base of support. In this study, a computed dynamic posturography system (Bertec Corporation, Columbus, Ohio, USA) was used to measure the postural and dynamic balance of the subjects. The balance system consists of a force plate mounted with force transducers and a display screen that provides visual feedback of the movements of the subject. The subjects were asked to shift their weight and lean in various directions instructed on the display as far and as fast as possible without losing balance. Composite scores were measured for movement velocity (LOS_MV), maximal excursion (LOS_ME), and directional control (LOS_DC). LOS_MV indicates the average speed of COP movement, while LOS_ME refers to the percentage distance moved toward the target on the initial movement. LOS_DC compares movement in the intended direction with extraneous movement when the participant is leaning toward the target (13). This balance system has yielded good test–retest reliability (ICC = 0.84–0.88) in people with stroke (14).

The BBS is used to assess balance when performing 14 different tasks, each of which is rated on a 5-point scale from 0 to 4. The total possible score of this scale is 56, with higher scores indicating better balance. This scale has shown excellent inter-rater and intra-rater reliability (ICC = 0.97–0.98) in people with stroke (15).

The TUG is a widely used assessment for measuring functional mobility and balance in people with stroke (16). It measures the total time a subject needs to complete multiple motor tasks, involving standing up from a chair, walking for 3 m, turning 180°, walking back, and sitting down. In people with stroke, the TUG has demonstrated excellent test–retest reliability (ICC = 0.95–0.97) and good concurrent validity with gait velocity (Spearman's ρ = −0.9, *p* < 0.01) and the 6-min walking test (Spearman's ρ = −0.960, *p* < 0.01) (16, 17).

The Cantonese version of the CIM assesses the extent of community integration of patients with chronic diseases. The CIM comprises 10 questions in four domains, namely, assimilation, support, occupation, and independent living (18). Each question is rated on a 5-point scale from 1, “always disagree,” to 5, “always agree.” If people with stroke have to compromise balance, executive function, and motor function, their walking ability would be affected correspondingly, which would contribute to the CIM score. The total possible CIM score is 50, with higher scores indicating a greater extent of community integration. The CIM has demonstrated good internal consistency, with the Cronbach's alpha values ranging from 0.79 to 0.83 (19).

### Statistical Analysis

Statistical Package for the Social Sciences (SPSS) software (version 27; IBM Corporation, Armonk, New York, United States) was used to conduct data analysis. The confidence level for significance was set at α = 0.05. Descriptive statistics were used to summarize the demographic data of participants. Normality of the data and homogeneity of the variances were assessed using the Shapiro–Wilk test and Levene's test, respectively. The independent *t*-test and the Mann–Whitney *U* test were used to compare parametric and nonparametric data between the subjects with stroke and healthy older adults, respectively.

ICCs were used to assess the inter-rater reliability (ICC_2, 1_) and test–retest reliability (ICC_3, 1_). Model 2 was chosen to measure the inter-rater reliability, as the raters were randomly assigned and generalization of results was allowed, while model 3 was chosen to assess the test–retest reliability, as the raters were fixed and a single measurement was taken. ICC values of <0.5, 0.5–0.75, 0.75–0.90, and >0.90 were considered to indicate poor, moderate, good, and excellent reliability, respectively (20).

To evaluate the validity, correlations of the TWT completion time with the FMA-LE, ankle muscle strength, LOS, BBS, TUG, and CIM scores were established using Pearson's *r* for all the parametric data and Spearman's rho for nonparametric data. Correlation (*r*) values <0.25, 0.25–0.49, 0.5–0.75, and >0.75 were considered to indicate no, fair, moderate to good, and good to excellent correlations, respectively (20).

The MDC indicates the minimal required change in test results to reflect an actual improvement in ability. It was calculated based on the test–retest reliability results by using the following formula: MDC = 1.96 × SEM × √2, where SEM = Sx √(1 – r) (21), Sx is the mean SD of the TWT results on days 1 and 2 and *r* is the test–retest reliability coefficient.

To determine the cutoff for the TWT completion time to distinguish between people with stroke and healthy older adults, the receiver operating characteristic (ROC) curve was plotted, with a trade-off between sensitivity and 1-minus specificity determined by Youden index. The area under the curve (AUC) was adopted to calculate discrimination accuracy. The AUC scores ≥0.9, 0.8 to <0.9, 0.7 to <0.8, 0.5 to <0.7, and ≤ 0.5 were considered to indicate outstanding, excellent, acceptable, poor, and no discrimination accuracy, respectively (20).

## Results

### Characteristics of the Participants

In total, 104 people with stroke and 53 healthy older adults were recruited in this study and their mean ages were 63.60 (SD 6.28) years and 61.74 (SD 7.39) years, respectively. The demographic characteristics of participants are shown in Table 1. The mean TWT completion time for people with stroke on day 1 (*n* = 104) (Rater A: 124.91 s, SD 87.20 s; Rater B: 124.40 s, SD 87.25) and day 2 (*n* = 69) (109.71 s, SD 60.60 s), the mean values of all other outcome measures, and the mean TWT completion time for healthy older adults (60.54 s, SD 15.00 s) are shown in Tables 2, 3. There is no significant difference in the baseline demographic characteristics between the subjects included in test–retest analysis and subjects included in correlation analysis (see Supplementary Material).

**Table 1:** Demographics of people with chronic stroke and healthy older adults.

**Parameter**	**People with chronic stroke (*n =* 104)**	**Healthy Older Adults (*n =* 53)**	***P*-value**
Age, years, mean (SD)	63.60 (6.28)	61.74 (7.39)	0.153
Sex, M/F, *n*	59/45	15/38	
Height, cm, mean (SD)	162.18 (11.57)	161.80 (8.48)	0.828
Weight, kg, mean (SD)	64.42 (9.72)	59.20 (11.36)	0.904
Body mass index, kg/m^2^, mean (SD)	25.24 (11.26)	22.52 (3.14)	0.41
Mobility status (unaided/stick/SBQ/LBQ /Rollator/Wheelchair), n	30/56/10/8/1/13	53/0/0/0/0/0	/
Stroke Type (Ischemic, Haemorrhagic), n	71/33	/	/
Dominant Side (Left/ Right), *n*	5/99	2/51	/
Hemi side (Left/ Right), *n*	45/59	/	/
Year since stroke, years, mean (SD)	6.66 (4.37)	/	/

*M, male; F, female; SBQ, small-based quadripod; LBQ, large-based quadripod*.

**Table 2:** Mean trail walking test (TWT) completion times for people with stroke and healthy older adults.

**Parameter**	**People with stroke**	**Healthy Older Adults (*n =* 53)**	**P-value**
Trail Walking test (Day 1 Rater A), s, mean (SD)	124.906 (87.2019) (*n =* 104)	60.54 (15.00)	*P* < 0.001
Trail Walking test (Day 1 Rater B), s, mean (SD)	124.404 (87.2496) (*n =* 104)	/	/
Trail Walking test (Day 2), s, mean (SD)	109.714 (60.5957) (*n =* 69)	/	/

**Table 3:** Mean Stroke-specific impairment outcome measurements for people with stroke.

**Parameter**	**People with stroke (*n =* 104)**
FMA-LE, mean (SD)	26.1 (4.50)
**Muscle strength—Ankle Dorsiflexor, kg, mean (SD)**	
Affected	11.14 (6.33)
Unaffected	16.60 (5.74)
**Muscle strength—Ankle Plantarflexor, kg, mean (SD)**	
Affected	8.62 (5.20)
Unaffected	13.40 (6.09)
LOS (RT), Affected, s, mean (SD)	1.49 (0.49)
LOS (RT), Unaffected, s, mean (SD)	1.41 (0.48)
LOS (MV), Affected, deg/s, mean (SD)	2.54 (1.51)
LOS (MV), Unaffected, deg/s, mean (SD)	2.67 (1.36)
LOS (end), Affected, %, mean (SD)	38.64 (20.32)
LOS (end), Unaffected, %, mean (SD)	45.62 (20.14)
BBS, mean (SD)	50.05 (6.83)
TUG, s, mean (SD)	17.42 (14.01)
CIM, mean (SD)	40.60 (7.05)

*FMA-LE, Fugl-Meyer assessment for lower limb; BBS, Berg Balance Scale; TUG, Timed Up and Go test; CIM, Community Integration Measure; LOS, Limit of stability; RT, reaction time; MV, movement velocity; End, endpoint/Max excursion*.

### Reliability and the MDC

The TWT completion time demonstrated excellent inter-rater reliability (ICC = 0.999) and good test–retest reliability (ICC = 0.876) in people with stroke (Table 4). The MDC in the TWT completion time was 19.02 s (Table 4).

**Table 4:** Inter-rater reliability, test-retest reliability of TWT completion time and minimal detectable change of subjects with stroke.

	**Test-retest—ICC_**3, 1**_**	**Inter-rater reliability—ICC_**2, 1**_**	**Minimal detectable change**
Trail walking test completion time	ICC = 0.876 (95%CI 0.733–0.935); *p* < 0.001	ICC = 0.999 (95%CI 0.999–1.000); *p* < 0.001	19.02

*CI, Confidence Interval*.

### Correlation of the TWT Completion Time With Other Outcome Measures

The correlations between the TWT completion time and the other outcome measures are shown in Table 5. The TWT completion time demonstrated a significant negative correlation with the FMA-LE scores (*r* = −0.409, *p* < 0.001), but no significant correlation with the ankle muscle strength measure. Furthermore, the TWT completion time showed a significant positive correlation with the LOS reaction time for the affected side (*r* = 0.256, *p* = 0.009) but no significant correlation with LOS reaction time for the unaffected side, and significant negative correlations with LOS movement velocity (affected and unaffected sides: *r* = −0.320, *p* < 0.001; *r* = −0.388, *p* < 0.001, respectively) and LOS endpoint excursion (affected and unaffected sides: *r* = −0.357, *p* < 0.001; *r* = −0.394, *p* < 0.001, respectively). In addition, the TWT completion time showed a moderate to good negative correlation with the BBS scores (*r* = −0.72, *p* < 0.001), a good to excellent positive correlation with the TUG completion time (*r* = 0.944, *p* < 0.001), but no significant correlation with the CIM scores.

**Table 5:** Concurrent validity of the TWT completion time for people with stroke and other outcome measures (*N* = 104).

	**Pearson's *r***	***p*-value**
FMA-LE	−0.409^**^	*P* < 0.001
Ankle dorsiflexion, unaffected, kg	−0.026	*p =* 0.791
Ankle dorsiflexion, affected, kg	−0.123	*p =* 0.213
ankle plantarflexion, unaffected, kg	−0.05	*p =* 0.614
Ankle plantarflexion, affected, kg	−0.13	*p =* 0.187
LOS (RT), affected, s	0.256	*p =* 0.009
LOS (RT), unaffected, s	−0.001	*p =* 0.995
LOS (MV), affected, deg/s	−0.320^**^	*p* < 0.001
LOS (MV), unaffected, deg/s	−0.388^**^	*p* < 0.001
LOS (end), affected, %	−0.357^**^	*p* < 0.001
LOS (end), unaffected, %	−0.394^**^	*p* < 0.001
BBS	−0.72^**^	*p* < 0.001
TUG, s	0.944^**^	*p* < 0.001
CIM	−0.032	*p =* 0.747

** *Correlation is significant at the 0.01 level (2 tailed). Abbreviations: FMA-LE, Fugl-Meyer assessment for lower extremity; BBS, Berg Balance Scale; TUG, Timed Up and Go test; CIM, Community Integration Measure; LOS, Limit of stability; RT, reaction time; MV, movement velocity; End, endpoint/Max excursion*.

### Cutoff Score

**Figure 3:**
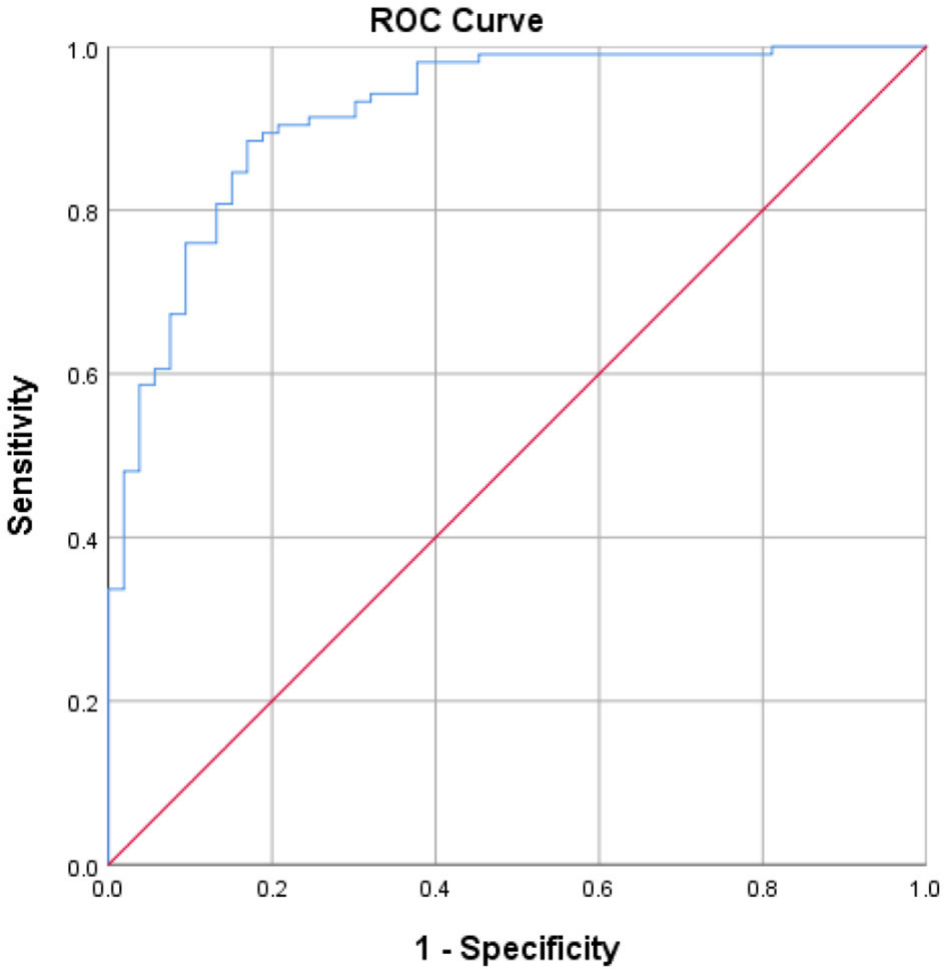
The receiver operating characteristic curves and the value of area under the receiver operating characteristic curve, sensitivity, and specificity for the optimal cutoffs of TWT completion time.

The optimal cutoff for the TWT completion time was found to be 69.61 s, with a sensitivity of 88.5%, a specificity of 83.0%, and the AUC of 0.919. This cutoff effectively differentiated between healthy older adults and people with stroke based on TWT performance. The Youden index analysis is shown in Figure 3.

## Discussion

### TWT Performance

This is the first study to extend the use of the TWT to assess the walking performance of community-dwelling people with stroke. The TWT performance of people with stroke in this study [stroke subjects: mean TWT completion time = 124.91 s; the Montreal Cognitive Assessment (MoCA) score ≤ 25] was found to be worse than that of older adults in a study by Klotzbier and Schott (mean TWT completion time < 60 s; MoCA score ≤ 25) (22). This difference between the two subject groups might be a result of greater physical limitations experienced by people with stroke than by people with probable mild cognitive impairment, such as a limited range of motion due to spasticity and abnormal gait pattern due to stroke, which increases the time required by affected subjects to rotate their body to search for and approach the next cone. Indeed, a previous study on body turning observed slower turning in people with stroke than in healthy adults (23). However, the contribution of physical and cognitive factors to TWT performance has not yet been established, and future research is needed to explore and differentiate the factors affecting TWT performance. Moreover, in the study by Klotzbier and Schott, subjects were instructed to perform five trials of the TWT (22); thus, the possible learning effect of the numbered route on repeated testing might have led to improved TWT performance among the subjects. Future research is needed to investigate the contribution of the learning effect to the TWT completion time.

Compared with the difference noted above, the TWT performance of our subjects with stroke (mean = 124.91 s) was closer to that of subjects with Down syndrome (DS) in another study (mean = 95.2 s) (24). However, the poor performance of people with DS was likely due to the high cognitive load on these subjects, who had an average age of 10.5 years and an IQ of 66.6 and would thus have faced difficulty in understanding test instructions. Even though the people with DS in the study by Klotzbier et al. were cognitively disadvantaged and younger, they still performed better on the TWT than our subjects with stroke. This finding implies that cognition and age play a lesser role than motor function in influencing TWT performance. The interplay between age, cognition, and motor function in TWT performance may be another area for future studies.

In this study, people with stroke (day 1: mean TWT complete time = 124.91 s) took, on average, more than twice as long as healthy older adults (mean = 60.54 s) to complete the TWT. This finding is consistent with that of Vive et al., who reported a lower comfortable gait speed in people with stroke vs. healthy older adults (mean = 0.93 vs. 1.4 m/s) (25). This may be because compared with healthy older adults, people with stroke have more physical limitations, such as spasticity, lower levels of muscle activation, and kinetic and coordination problems, which have a direct influence in reducing the gait speed (26). Furthermore, the sequential linking of numbers (1–15) also entails cognitive functions during the TWT. Compared with healthy older adults, people with stroke may suffer from various cognitive problems due to different pathologies, such as lower processing speed, impaired executive ability, and poor working memory (27).

The TWT performance of healthy older adults in this study (mean TWT complete time = 60.54 s) is comparable to that in study by Yamada and Ichihashi (3) (non-falling older adults: mean = 61.5 s). This comparability may be due to the standardized instructions and similar test designs adopted in both studies. However, the TWT performance of our healthy older adults was slower than that of the healthy older adults in a study by Klotzbier and Schott (22) (mean age = 68.2 ± 6.42 years, mean TWT complete time = ~41 s). This difference may be due to the longer total walking distance used in this study (5 m × 5 m in this study vs. 4 m × 4 m in the study by Klotzbier and Schott).

### Reliability of the TWT for Assessing People With Stroke

This was the first study to investigate the reliability of the TWT for assessing people with stroke. Our results revealed that the TWT has excellent inter-rater reliability (ICC = 0.999–1) and good test–retest reliability (ICC = 0.756–0.939), indicating high reliability for accessing motor–cognitive function in people with stroke. The research personnel was well-trained in performing the standardized protocol, providing explicit instructions, and taking consistent measurements, which might have reduced measurement errors and contributed to excellent inter-rater reliability. The good test–retest reliability suggests that the 7-day test–retest interval adopted in this study is suitable for minimizing learning effects and preventing changes in the conditions of subjects.

### Comparing Inter-rater Reliability of the TWT With That Reported in the Literature

So far, no study has systematically investigated the inter-rater reliability of the TWT in people with stroke. We found that the results of the TWT in this study on people with stroke (ICC = 0.999–1) are consistent with those of other motor–cognitive tests, including walking with a cognitive component (quoting animals) and the Walking and Remembering Test (ICC range = 0.8–0.99) in subjects with the Parkinson's disease (28) and healthy older adults (29).

### Comparing Test–Retest Reliability of the TWT With That Reported in the Literature

Consistent with previous studies of the TWT in frail community-dwelling older adults (3), older adults with probable mild cognitive impairment (22), and children with DS (24), this study demonstrated good to excellent test–retest reliability of the TWT in people with stroke (ICC = 0.756–0.939). While the study of Yamada showed excellent test–retest reliability of the TWT (ICC = 0.945), the only difference between the study of Yamada and this study lies in the test–retest interval (3). Yamada performed the retest 2 weeks after the first trial, while we performed the retest 7 days after the first trial (3). Despite this difference in the test–retest interval, the good to excellent results obtained consistently in this study suggest the high reliability of the TWT for use in people with stroke. Studies by Klotzbier also demonstrated a higher test–retest reliability of the TWT (ICC = 0.83–0.97 and 0.861–0.916, respectively) than that in this study (22, 24). However, this difference was expected, as the retests in both studies by Klotzbier were conducted within a single session, in which subjects with probable mild cognitive impairment completed five trials and children with DS completed three trials of the TWT with resting intervals. These small test–retest intervals might have resulted in a significant learning effect and thus little variance in the results between the trials.

The difference in TWT completion times (~64.4 s) between people with stroke and healthy older adults in this study markedly surpassed the calculated MDC (time: 19.02 s). This between-group disparity suggests that there was a genuine difference rather than a measurement error.

### Correlation Between the TWT Completion Time and Other Outcome Measures

The FMA-LE is used to evaluate motor function impairment and reflexes in the lower extremities of people with stroke (8). A significant fair correlation was found between the TWT completion time and FMA-LE scores (*r* = −0.409, *p* < 0.001). The FMA-LE scores reflect neural control, including postural timing, and muscle performance, such as hip-knee extension and ankle dorsiflexion, which are essential components of good walking quality. Hence, it is expected that individuals with a higher FMA-LE score would achieve a shorter TWT completion time.

Surprisingly, no significant correlation was found between the ankle muscle strength and TWT completion time. Previous studies have reported that the muscle groups in the ankles, especially the plantar flexors, play an important role in regulating gait speed by providing energy to propel lower limb movement during the push-off phase (30). A possible explanation for this lack of correlation is that the TWT is a type of motor–cognitive test. Thus, apart from physical factors, cognitive factors may influence the performance of people with cognitive impairments on this test (24). For example, subjects were required to walk past 15 numbered plastic cones sequentially (1–15) during walking in the TWT, a task that requires both the working memory and executive function. However, further studies are needed to investigate the extent to which cognitive and motor components contribute to the TWT completion time.

Unexpectedly, the three components of the LOS test (RT, MV, and end) showed only significant fair correlations with the TWT completion time. A possible explanation is the different focus of balance between the two measures. The LOS test assesses the ability of subjects to shift their COP without changing the base of support or losing balance, which requires the subjects to stand in a double-limb stance on a force plate. In contrast, the TWT requires subjects to walk and turn, which involves the shifting of their COP in a single-limb stance (31). Thus, the LOS test only focuses on the static balance of subjects in a laboratory setting, whereas the TWT challenges their dynamic balance more, as they are required to walk and select numbered cones, a task that elicits motor–cognitive functions required in daily tasks, such as shopping.

The total BBS score showed a strong negative correlation with the TWT completion time (*r* = −0.72, *p* < 0.001). This strong correlation could be due to the inclusion of similar motor tasks in these two tests, such as turning and looking behind when considering the numbered cones, turning 360° when walking to the next cone and standing unsupported. In addition, the BBS, which is used to evaluate the static and dynamic balance of subjects, has been found to demonstrate a moderate correlation with the Dynamic Walking Index (*r* = 0.75–0.77; *p* < 0.01), which is used to evaluate the walking function in older people with balance problems (32). This correlation indicates that subjects with higher BBS scores should have better walking stability and quality, which is directly correlated with a shorter TWT completion time.

A significant good to excellent correlation was found between the TWT completion time and TUG completion time (*r* = 0.944, *p* < 0.001), which is consistent with the findings of a previous study on community-dwelling older adults (*p* = 0.001, correlation significant at *p* < 0.01) (3). The significant correlation between these two tests could be due to the assessment of the balance of participants and locomotion in walking and turning in both these tests. However, TWT is an assessment tool developed for evaluating both motor and cognitive function. The unexpected high correlation between TWT and TUG may reflect that domain of cognitive function make a minor contribution during the TWT. The cognitive function is high among our recruited subjects as screened by the inclusion criteria of AMT ≥7. Good cognitive function of our subjects allows them to handle the dual-task ambulation without significant difficulty. In order to investigate the reliability and validity of TWT, more subjects with different levels of cognitive function would be recruited in future studies.

An insignificant correlation was found between the TWT completion time and CIM scores (*r* = −0.032, *p* = 0.747). One possible explanation is the difference in measurement domains between these two tests. The CIM is a subjective measurement of community integration that considers the self-reported subjective feelings of participants (18). In contrast, the TWT is an objective measurement of motor–cognitive dual tasking in a laboratory setting, which may not reflect the subjective feelings of participants in real-life situations. Furthermore, previous studies have proven that motor function alone is insufficient in predicting the community reintegration of people with stroke. Other factors, including poststroke depression, personal perceptions, and stroke recovery, are also important predictors of community reintegration in this population (*r* = 0.499–743, *p* < 0.01) (33, 34).

### Cutoff Scores of the TWT

The TWT completion times were well differentiated between subjects with stroke and healthy older adults in this study (AUC = 0.919; Figure 3). The high AUC indicates excellent accuracy of the TWT in discriminating between these two groups. Using the Youden index (0.715), the TWT completion time cutoff to differentiate between these groups was found to be 69.61 s, with a high sensitivity of 88.5% and a specificity of 83.0%. Thus, the TWT is a sensitive and specific test that can identify people with stroke who have impaired dual-tasking ambulation ability with an outstanding diagnostic power.

### Limitations and Further Study

Several limitations of this study need to be acknowledged. First, although this study investigated the performance of subjects in the TWT, the quality of their movements and the number of errors they made while performing the TWT were not considered, as the TWT completion time was the focus of this study. Therefore, future studies are suggested to assess the correlation between the number of errors made in the TWT and other assessments. Second, as the TWT is a motor–cognitive dual task, both the physical and cognitive limitations may affect the TWT performance of subjects. It would be worth investigating the extent to which cognitive and physical limitations contribute to poor TWT performance in people with stroke. Third, the literature reports varying instructions for administering the TWT. For example, in study by Klotzbier, cones were positioned inside a grid with an area of 4 m^2^ and subjects performed 5 trials (22). In this study, a grid with an area of 5 m^2^ and 1 trial were used. Our hypothesis that the learning effect of the TWT trial pattern and a smaller test area improve the performance of subjects that warrants further investigation and such research could help to develop standardized instructions for TWT implementation and interpretation. Fourth, total 69 and 104 people with stroke participated in the reliability and correlation analyses, respectively. The sample size estimation was based on the effect sizes calculated for reliability and correlation analyses of TWT. Larger sample size should be included in the future study to draw a more robust conclusion. Fifth, our subjects were recruited from the local self-help group and they have a relatively higher cognitive function with Abbreviated Mental Test scores ≥7. Subjects with a lower cognitive function should be included in the future study, so that our results could be generalized to the general stroke population. Lastly, as the incidence of stroke is associated with age, with its incidence doubles each decade after 55 years (35), only subjects ≥45 years old were recruited in this study. Thus, our result could only apply to those subjects who fulfilled our inclusion criteria. The TWT could be extended to those who are younger and with more severe cognitive impairments so as to further enhance the validity of TWT in the general stroke population in the future work.

## Conclusion

Results of this preliminary study demonstrated that TWT is a reliable, valid, sensitive, and specific clinical test for evaluating dual-tasking ambulation ability in people with stroke aged 45 years or above and without cognitive impairments. The completion time of TWT can differentiate the dual-tasking ambulation ability between people with stroke and healthy older adults. As the reliability and validity of the TWT are found in patients with stroke without cognitive impairments, the added value of this test should be tested in a population with more severe cognitive impairment.

## Data Availability Statement

The raw data supporting the conclusions of this article will be made available on reasonable request by the corresponding author.

## Ethics Statement

The studies involving human participants were reviewed and approved by the Departmental Research Committee of The Hong Kong Polytechnic University. The patients/participants provided their written informed consent to participate in this study.

## Author Contributions

SN contributed to the conception and design of the study. PC, TC, MC, AL, LN, and KS collected the data and organized the database. T-WL, TC, MC, AL, LN, and KS performed the statistical analysis. SN, T-WL, JT, MT, TC, MC, AL, LN, and KS wrote the first draft of the manuscript. All the authors contributed to manuscript revision, read, and approved the submitted version of the manuscript.

## Funding

This study is supported by the General Research Fund (Ref: 15101217) from the Research Grant Council, Hong Kong, to SN and her team.

## Conflict of Interest

The authors declare that the research was conducted in the absence of any commercial or financial relationships that could be construed as a potential conflict of interest.

## Publisher's Note

All claims expressed in this article are solely those of the authors and do not necessarily represent those of their affiliated organizations, or those of the publisher, the editors and the reviewers. Any product that may be evaluated in this article, or claim that may be made by its manufacturer, is not guaranteed or endorsed by the publisher.
