# Supplementary material for: Psychometric Properties of the Trail Walking Test for People With Stroke
Source: Front Neurol. 2022 Mar 3;13:821670. doi: 10.3389/fneur.2022.821670 (PMC8929289; doi:10.3389/fneur.2022.821670)
Supplement: Supplementary file 1 [file Table_1.DOCX]

| Parameter | People included in the correlation analysis (n=104) | People included in the test-retest reliability analysis (n=69) | P value |
| --- | --- | --- | --- |
| Age, years, mean (SD) | 63.60 (6.28) | 63.65 (6.51) | 0.955 |
| Sex, M/F, n | 59/45 | 41/28 | 0.726 |
| Height, cm, mean (SD) | 162.18 (11.57) | 162.25 (13.40) | 0.973 |
| Weight, kg, mean (SD) | 64.42 (9.72) | 65.05 (10.43) | 0.692 |
| Body mass index, kg/m2, mean (SD) | 25.24 (11.26) | 25.88 (13.84) | 0.746 |
| Mobility status (unaided/stick/SBQ/LBQ/Rollator/Wheelchair), n | 30/56/10/8/1/13 | 23/37/6/2/0/8 | 0.757 |
| Stroke Type (Ischemic, Haemorrhagic), n | 71/33 | 47/22 | 0.983 |
| Dominant Side (Left/ Right), n | 5/99 | 4/65 | 0.774 |
| Hemi side (Left/ Right), n | 45/59 | 31/38 | 0.830 |
| Year since stroke, years, mean (SD) | 6.66 (4.37) | 7.35 (4.73) | 0.325 |
| Trail Walking test (Day 1 Rater A), s, mean (SD) | 124.68 (87.20) | 114.93 (56.33) | 0.412 |
